# Ca^2+^/Calmodulin-Dependent Protein Kinase II and Androgen Signaling Pathways Modulate MEF2 Activity in Testosterone-Induced Cardiac Myocyte Hypertrophy

**DOI:** 10.3389/fphar.2017.00604

**Published:** 2017-09-11

**Authors:** Javier Duran, Daniel Lagos, Mario Pavez, Mayarling F. Troncoso, Sebastián Ramos, Genaro Barrientos, Cristian Ibarra, Sergio Lavandero, Manuel Estrada

**Affiliations:** ^1^Programa de Fisiología y Biofísica, Instituto de Ciencias Biomédicas, Facultad de Medicina, Universidad de Chile Santiago, Chile; ^2^Advanced Center for Chronic Diseases (ACCDiS), Facultad Ciencias Químicas y Farmacéuticas and Facultad Medicina, Universidad de Chile Santiago, Chile; ^3^Department of Internal Medicine (Cardiology Division), University of Texas Southwestern Medical Center, Dallas TX, United States

**Keywords:** CaMKII, MEF2C, testosterone, androgen receptor, cardiac myocyte, hypertrophy

## Abstract

Testosterone is known to induce cardiac hypertrophy through androgen receptor (AR)-dependent and -independent pathways, but the molecular underpinnings of the androgen action remain poorly understood. Previous work has shown that Ca^2+^/calmodulin-dependent protein kinase II (CaMKII) and myocyte-enhancer factor 2 (MEF2) play key roles in promoting cardiac myocyte growth. In order to gain mechanistic insights into the action of androgens on the heart, we investigated how testosterone affects CaMKII and MEF2 in cardiac myocyte hypertrophy by performing studies on cultured rat cardiac myocytes and hearts obtained from adult male orchiectomized (ORX) rats. In cardiac myocytes, MEF2 activity was monitored using a luciferase reporter plasmid, and the effects of CaMKII and AR signaling pathways on MEF2C were examined by using siRNAs and pharmacological inhibitors targeting these two pathways. In the *in vivo* studies, ORX rats were randomly assigned to groups that were administered vehicle or testosterone (125 mg⋅kg^-1^⋅week^-1^) for 5 weeks, and plasma testosterone concentrations were determined using ELISA. Cardiac hypertrophy was evaluated by measuring well-characterized hypertrophy markers. Moreover, western blotting was used to assess CaMKII and phospholamban (PLN) phosphorylation, and MEF2C and AR protein levels in extracts of left-ventricle tissue from control and testosterone-treated ORX rats. Whereas testosterone treatment increased the phosphorylation levels of CaMKII (Thr286) and phospholambam (PLN) (Thr17) in cardiac myocytes in a time- and concentration-dependent manner, testosterone-induced MEF2 activity and cardiac myocyte hypertrophy were prevented upon inhibition of CaMKII, MEF2C, and AR signaling pathways. Notably, in the hypertrophied hearts obtained from testosterone-administered ORX rats, both CaMKII and PLN phosphorylation levels and AR and MEF2 protein levels were increased. Thus, this study presents the first evidence indicating that testosterone activates MEF2 through CaMKII and AR signaling. Our findings suggest that an orchestrated mechanism of action involving signal transduction and transcription pathways underlies testosterone-induced cardiac myocyte hypertrophy.

## Introduction

The biological effects of androgens on cardiac myocytes are tightly regulated by the circulating plasma levels of testosterone, the specific activation of intracellular signaling networks by this hormone, and, consequently, the functional integration of these signaling pathways. Currently, there is growing interest in elucidating the mechanisms involved in androgen-mediated cardiac regulation because exogenous testosterone administration in various interventional studies has been found to produce improvements in age-related and muscular disorders that affect males (Handelsman and Liu, 2005; Kapoor et al., 2007; Ikeda et al., 2009; Cook and Romashkan, 2011). Testosterone at normal concentrations is required for inducing diverse physiological effects in cardiac myocytes (English et al., 2000; Kloner et al., 2016), but abnormalities in plasma testosterone concentrations cause cardiovascular diseases (Malkin et al., 2003; Oskui et al., 2013; Ayaz and Howlett, 2015; Kloner, 2016). Therefore, the therapeutic use of testosterone is limited mainly by the potential adverse cardiovascular effects (Liu et al., 2003; Allameh et al., 2016). An after-effect produced by elevated circulating levels of testosterone is cardiac hypertrophy; although the initial growth of cardiac myocytes that is triggered here is an adaptive response, a sustained anabolic environment becomes detrimental and leads to an increase in cardiac morbidity and mortality (Thum and Borlak, 2002; Liu et al., 2003). However, most of this evidence was gathered from studies on people who abused anabolic steroids or were administered multiple drugs, and this has precluded a clear understanding of the precise actions of androgens; thus, the mechanisms underlying testosterone signaling in cardiac myocytes remain largely elusive.

Testosterone exerts its effects mainly by activating the androgen receptor (AR) (Heemers and Tindall, 2007; Claessens et al., 2008). AR-regulated transcription is mediated either directly by the activated AR or by the receptor in association with additional transcription factors and coregulator proteins in a cell-specific manner (Mooradian et al., 1987; Tan et al., 2015). Moreover, testosterone activates cell signaling cascades that have been postulated to participate in physiological and hypertrophic effects on cardiac myocytes (Vicencio et al., 2006; Altamirano et al., 2009). Hypertrophic agonists have been widely reported to elevate intracellular Ca^2+^ levels and stimulate Ca^2+^-dependent pathways in cardiac myocytes (Hefti et al., 1997; Zarain-Herzberg et al., 2011). Previously, we showed that testosterone induces an increase in intracellular Ca^2+^ in cardiac myocytes (Vicencio et al., 2006), in which the integration of the Ca^2+^ signals through cytosolic kinases modulates androgen actions. Ca^2+^/calmodulin-dependent protein kinase II (CaMKII), a cellular decoder of Ca^2+^ signals, plays a central role in cardiac myocytes by directing extracellular signaling to downstream transcription factors (Passier et al., 2000; Zhang et al., 2007; Bossuyt et al., 2008; Anderson et al., 2011). The activity of CaMKII is controlled principally by the binding of the Ca^2+^/calmodulin complex and the autophosphorylation of CaMKII at Thr286/287 (Hoch et al., 1999). CaMKII expression and activity are altered in cardiac hypertrophy and heart failure (Ramirez et al., 1997), and the overexpression of CaMKIIδ, the principal isoform detected in heart, increases the mRNA levels of β-myosin heavy chain (β-MHC), α-skeletal actin (SKA), atrial natriuretic peptide, and brain natriuretic peptide, which are all well-characterized cardiac hypertrophy markers (Ramirez et al., 1997; Zhang et al., 2007). Conversely, CaMKII inhibition prevents cardiac hypertrophy triggered by adrenergic agonists (Zhu et al., 2000). Once CaMKII is activated, it phosphorylates and induces the nuclear export of the transcriptional repressor class II histone deacetylase type 4/5 (HDAC 4/5); this, in turn, triggers the activation of MEF2 (Backs et al., 2006, 2008; Zhang et al., 2007, 2010), a member of the MADS (MCM-1, Agamous, Deficiens, Serum response factor)-box-family of transcription factors that regulate cardiac myocyte growth and differentiation. MEF2 contains a MADS-box domain at the N-terminus that dimerizes and binds to the cognate DNA sequence [CAT(A/T)_4_TAG/A] in the promoter regions of target genes (Black and Olson, 1998; Zhang et al., 2010).

MEF2-regulated transcription performs fundamental functions by controlling gene-expression programs involved in the differentiation of cardiac myocytes (Lin et al., 1997; McKinsey et al., 2002; McKinsey and Olson, 2005); in this transcriptional control, the MEF2 MADS domains mediate binding to the promoter regions of MEF2-regulated genes (Potthoff and Olson, 2007). Furthermore, various hypertrophic agonists activate MEF2 and thereby induce cardiac myocyte hypertrophy, and one specific MEF2 isoform, MEF2C, functions as a mediator of cardiac and skeletal muscle differentiation and growth (Molkentin et al., 1996; Zhang et al., 2007; Munoz et al., 2009). In human skeletal muscle, AR-binding regions in several genes were reported to be enriched in MEF2C-binding sequences and thus potentially under MEF2C transcriptional control (Wyce et al., 2010), and MEF2C was suggested to modulate numerous AR targets and androgen-responsive genes involved in muscle growth: Chip-qPCR experiments revealed that androgen treatment of muscle cells increased AR binding to these genes that are potentially regulated by MEF2C (Wyce et al., 2010). Therefore, this previous study suggested that coordinated transcriptional mechanisms regulate the expression of target genes shared by AR and MEF2C. However, limited evidence is available regarding the intracellular pathways modulated by testosterone in cardiac myocytes under either normal or hypertrophic conditions, and it remains unclear whether similar pathways are modulated at the organ level.

Because the AR and the MEF2 pathways of transcriptional regulation both control gene-expression programs related to cell growth—a crucial requisite for the induction of cardiac hypertrophy—we investigated whether these pathways are linked in testosterone-induced cardiac myocyte hypertrophy. In this study, we determined that testosterone activates MEF2 through CaMKII and AR signaling. Our findings suggest a cooperative mechanism of androgen action involving signal transduction and transcription pathways in cardiac myocyte hypertrophy.

## Materials and Methods

### Materials

The following reagents were from commercial sources: testosterone, bicalutamide, AIP, and 5-bromo-2-deoxyuridine (BrdU), Sigma-Aldrich (St. Louis, MO, United States); anti-phospho-CaMKII (Thr286, cat. # 3361), anti-MEF2C (cat. # 5030) and anti-phospholamban (PLN, cat. # 14562) antibodies, Cell Signaling Technology (Danvers, MA, United States); CellTracker Green (5-chloromethyl fluorescein diacetate) from Thermo-Fisher Scientific (Rockford, IL, United States); anti-phospho-PLN (Thr17, cat. # sc-24565), anti-CaMKII (cat. # sc-5392) and anti-AR (cat. # sc-815) antibodies and siRNAs targeting CaMKIIδ (cat. # sc-38953), AR (cat. # sc-29204), and MEF2C (cat. # sc-38062), Santa Cruz Biotechnology (Santa Cruz, CA, United States); [^3^H]-leucine, NEN Radiochemicals Perkin Elmer (Waltham, MA, United States); and collagenase type II, Worthington Biochemical Corporation (Lakewood, CA, United States). All other reagents were of analytical grade and are commercially available.

### Culture of Neonatal Rat Cardiac Myocytes

This study was carried out in accordance with the recommendations of the National Institutes of Health Guide for the Care and Use of Laboratory Animals. The protocol was approved by the local Institutional Animal Care and Use Committee of the Faculty of Medicine, University of Chile (protocol CBA # 0768 FMUCH). Rats were bred in the Animal Breeding Facility of the Faculty of Medicine, University of Chile. Primary cultures of neonatal cardiac myocytes were prepared from the hearts of 1–3-day-old Sprague-Dawley rats, as described previously (Ibarra et al., 2013). The use of this protocol yields cardiac myocyte cultures that are at least 90% pure, and these cultures represent an established cellular model for studying cardiac hypertrophy (Chlopcikova et al., 2001). To prevent fibroblast growth, the growth medium was supplemented with 2.5 μM BrdU. Cardiac myocytes were cultured in growth medium containing DMEM:M-199 (4:1) supplemented with 10% FBS and 1% penicillin-streptomycin, and were deprived of FBS for 24 h before hormone stimulation.

### Cardiac Hypertrophy *In Vivo*

Twelve, 8-week-old, male Sprague-Dawley rats (body weight ∼300 g) were ORX. Additionally, six normal rats without testosterone treatment were used as control animals. For surgery, animals were anesthetized with ketamine (70 mg⋅kg^-1^, ip) and xylazine (7 mg⋅kg^-1^, ip), and after the surgery, the ORX rats were administered tramadol (0.05 mg⋅kg^-1^, sc) for pain control and allowed to recover for 7 days before testosterone supplementation. The animals were allowed free access to food and water and were maintained under a 12/12-h light/dark cycle. The ORX rats were randomly assigned to two groups (*n* = 6 each): ORX plus vehicle (peanut oil) treatment; and ORX plus supplementation with testosterone (125 mg⋅kg^-1^⋅week^-1^) for 5 weeks. Normal rats treated with vehicle served as the control group. Plasma testosterone concentrations were evaluated using ELISA (Cayman Chemical, Ann Arbor, MI, United States). After the treatment, the ORX and control rats were weighed and then euthanized by administering an overdose of sodium pentobarbital (200 mg⋅kg^-1^), after which the hearts were dissected and weighed to calculate the left-ventricle and heart weight ratio with respect to body weight and tibia length. Moreover, seminal vesicles and prostates were weighed to evaluate systemic effects of the administrated testosterone.

### Transient Transfection and Reporter-Gene Assays

MEF2 transcriptional activity was evaluated by using the plasmid 3XMEF2-Luc (Addgene plasmid #32967), which contains MEF2-binding boxes cloned upstream of the firefly luciferase reporter gene; 3XMEF2-Luc was a gift from Dr. Ron Prywes. Furthermore, cardiac myocytes were transfected with either a plasmid expressing a wild-type isoform of CaMKII (XE117 CAMKII-CS2+; Addgene plasmid #16737), or a plasmid expressing a constitutively active form of CaMKII (XE118 CAMKII-T286D-CS2+; Addgene plasmid #16736). In this active form of CaMKII, Thr286 is mutated to Asp, which mimics the phosphorylation of this site and results in CaMKII activation independently of binding to Ca^2+^/calmodulin; XE118 CAMKII-T286D-CS2+ was a gift from Dr. Randall Moon. A plasmid expressing *Renilla* luciferase was used as the control for transcriptional activity (Promega, Madison, WI, United States). Transfections were performed using Lipofectamine 2000 (Invitrogen, Carlsbad, CA, United States), according to manufacturer specifications, and the plasmid DNA was used at a final concentration of 1 μg⋅mL^-1^ in each experimental condition. Cardiac myocytes were incubated with testosterone for 24 h in the presence or absence of inhibitors, and then the cells were lysed and MEF2-Luc and *Renilla* luciferase activities were measured after 24 h of testosterone stimulation, to allow accumulation of gene product (Wu et al., 2006), using the dual-luciferase kit Assay Reporter System (Promega, Madison, WI, United States) and a luminometer (Berthold luminometer F12, Pforzheim, Germany).

In addition to the inhibitor experiments, we performed knockdown experiments by transfecting cardiac myocytes with siRNAs specifically targeting CaMKIIδ (siRNA-CaMKIIδ), MEF2C (siRNA-MEF2C), and AR (siRNA-AR). As a control, cardiac myocytes were transfected with a non-targeting siRNA (Control siRNA-A; Santa Cruz Biotechnology, sc-37007). For this set of experiments, cardiac myocytes grown on 60-mm dishes were transfected with 20 nM siRNAs by using Lipofectamine 2000, and then protein downregulation in each experimental condition was confirmed through Western blotting.

### Real-time PCR

For mRNA-expression analysis, total RNA was isolated from lysates prepared from homogenized left-ventricle tissue of both normal and ORX rats; lysates were prepared using TRIzol^®^ reagent (Invitrogen, Carlsbad, CA, United States). Next, 2 μg of the isolated RNA was reverse-transcribed in a reaction volume of 20 μL containing 1 μM Oligo-dT primer, 0.5 μM dNTPs, 10 U of RNase inhibitor, and SuperScript II Reverse Transcriptase (Thermo-Fisher Scientific, Rockford, IL, United States), according to the manufacturer’s instructions. Quantitative real-time PCR (qPCR) was performed using the StepOnePlus^TM^ Real-Time PCR System (Applied Biosystems, Foster City, CA, United States); at least three independent qPCR experiments were performed for each time point. The following primer sequences were used: β-MHC: 5′-AAGTCCTCCCTCAAGCTCCTAAGT-3′, 5′-TTGCTTTGCCTTTGCCC-3′; GAPDH: 5′-ACATGCCGCCTGGAGAAAC-3′, 5′-AGCCCAGGATGCCCTTTAGT-3′. Expression values were normalized relative to the mRNA levels of GAPDH, used as the internal control, and are reported in units of 2-ΔΔCT ± SE. The CT values were determined by using MXPro software in cases where the fluorescence was 25% higher than background. PCR products were verified using melting-curve analysis.

### Immunocytochemistry

Immunofluorescent labeling was performed as described previously (Ibarra et al., 2013). Briefly, cardiac myocytes were stimulated with testosterone (100 nM) for different times, fixed with paraformaldehyde (4%), and then incubated with rabbit anti-MEF2C antibody (1:400; 4°C, overnight). Next, the cells were washed thrice with PBS, and then incubated with goat anti-rabbit Alexa Fluor 488 IgG (H + L) (1:500; room temperature, 1 h), after which nuclei were counterstained with 4′-6-diamidino-2-phenylindole (DAPI) for 10 min. Images were acquired using a fluorescence microscope (Colibri.2 LED system, Carl Zeiss AG, Oberkochen, Germany) and analyzed using ImageJ software (NIH, Bethesda, MD, United States). To quantify fluorescence, the summed pixel intensity was calculated for each section delimited by a region of interest (ROI) within either the nucleus or the cytoplasm of cardiac myocytes that had been stimulated or not stimulated with testosterone.

### Protein Extraction and Western Blotting

Lysates were prepared from cardiac myocytes that were grown on 60-mm plates and serum-starved for 24 h before exposure to testosterone for various times (indicated in Results and in figure legends). Cells were washed with ice-cold PBS and scraped off plates in 70 μl harvesting lysis buffer [150 mm NaCl, 1 mm EGTA, 1% Nonidet P-40, 50 mm Tris-HCl (pH 7.4), 5 mm sodium orthovanadate, 20 mm NaF, 1 μg/ml aprotinin, 1 μm pepstatin, 20 μm leupeptin, 1 mM benzamidine, and 0.2 mm 4-(2-aminoethyl)benzene sulfonyl fluoride)]. Furthermore, tissue lysates were prepared by homogenizing left-ventricle samples and centrifuging them at 15,000 ×*g* for 10 min. Supernatants were removed, divided into portions and protein concentrations were determined by using a bicinchoninic acid assay kit according the manufacturer’s instructions (Thermo-Fisher Scientific, Rockford, IL, United States). Equal amounts of proteins (20 μg) were denatured at 100°C in 30% glycerol, 8% sodium dodecyl sulfate, 10% 2-mercaptoethanol, 25 mm Tris-HCl (pH 6.8), and 0.1% bromophenol blue; resolved by 10 or 15% (for PLN) acrylamide-bisacrylamide gels and transferred to nitrocellulose membranes as previously described (Altamirano et al., 2009). Membranes were blocked in 5% bovine serum albumin in Tris-buffered saline containing 0.05% Tween 20 and then immunoblotted with primary antibodies (1:1000). The protein bands in the blots were visualized using the ECL detection kit Westar Supernova (Cyanagen, Bologna, Italy), and band intensities were determined through densitometric scanning with ImageJ software.

### Cell-Size Measurements

Transfected and non-transfected cardiac myocytes were cultured on gelatin-coated coverslip for 24 h, and then the culture medium was replaced with a medium supplemented with testosterone together with or without inhibitors and the cells were cultured for another 48 h. For cell-size measurement, cardiac myocytes were incubated with the vital fluorescent dye CellTracker Green for 45 min, after which fluorescence images were acquired (using a Colibri.2 LED illumination system, Zeiss) and analyzed and compared using ImageJ software. For the measurements, we used at least eight different fields from five independent cultures in each condition (>100 cells). Cell size measurements were performed blinded.

### Amino Acid Incorporation

Cardiac myocytes were cultured for 24 h and then incubated with [^3^H]-leucine (2.5 μCi⋅mL^-1^) for an additional 48 h in the presence or absence of testosterone (Duran et al., 2016). Next, the cells were washed four times with ice-cold PBS and treated with 10% trichloroacetic acid at 4°C for 60 min. The samples were centrifuged for 20 min at 15,000 ×*g*, and the obtained pellets were washed once with ice-cold absolute acetone and dissolved in 0.2 M NaOH. Aliquots of triplicate samples per group were counted in a liquid scintillation counter (Beckman Instruments, Fullerton, CA, United States).

### Statistical Analysis

All data are expressed as means ± SEM and presented as fold-induction relative to the control in each experiment. Sample size for each experimental procedure were calculated by *a priori* power analyses using the G^∗^Power 3 software (Faul et al., 2007). Statistical analysis was performed using *t*-tests or ANOVA for multiple comparisons, followed by a *post hoc* Bonferroni test. In each figure, “*n*” indicates the number of independent experiments performed on different cell cultures or animal groups. *P* < 0.05 was considered statistically significant. All analyses were conducted using GraphPad Prism 5 software (GraphPad Software, San Diego, CA, United States).

## Results

### Testosterone Activates CaMKII in Cardiac Myocytes

**FIGURE 1:**
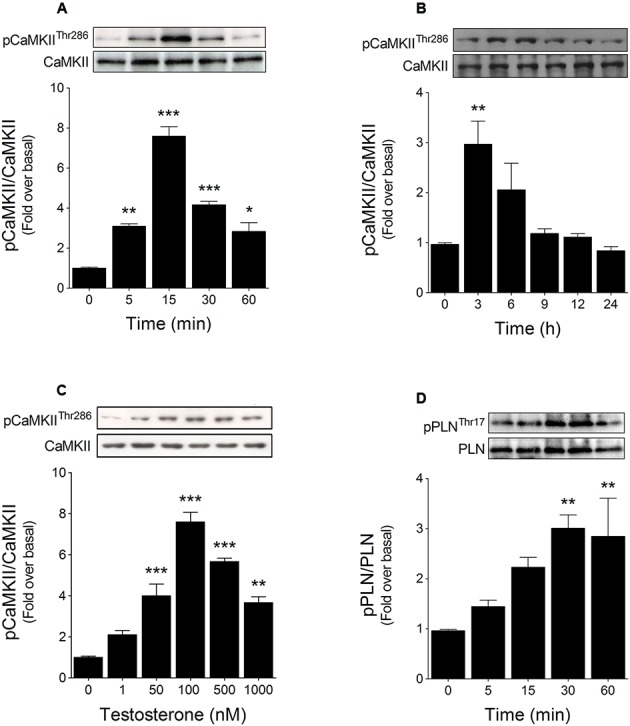
Testosterone activates CaMKII in cardiac myocytes. Cardiac myocytes were stimulated with testosterone and then subjected to Western blotting to determine the levels of phosphorylated CaMKII (pCaMKII) and phospholamban (pPLN). CaMKII phosphorylation kinetics were assessed over short and long periods of testosterone stimulation: CaMKII phosphorylation was evaluated from **(A)** 5 to 60 min (*n* = 5) and **(B)** 3 to 24 h (*n* = 6). **(C)** Cardiac myocytes were stimulated for 15 min with the indicated concentrations of testosterone (*n* = 6). **(D)** PLN Thr17 phosphorylation and total protein levels in cells treated for 5–60 min with 100 nM testosterone (*n* = 5). The results of densitometric analyses are shown as ratios of phosphorylated to total protein. Data are presented as means ± SEM. *P*-values were determined using ANOVA followed by Bonferroni *post hoc* test; ^∗^*P* < 0.05, ^∗∗^*P* < 0.01, ^∗∗∗^*P* < 0.001 vs. control.

CaMKII phosphorylation at Thr286 is a well-established parameter for monitoring CaMKII activity (Ramirez et al., 1997; Hughes et al., 2001). Previously, we showed that testosterone treatment induced a rapid (<30 min) increase in CaMKII phosphorylation in cardiac myocytes (Wilson et al., 2013). To extend these findings, we examined the kinetics of CaMKII phosphorylation over short (5–60 min) and long (3–24 h) periods of stimulation with 100 nM testosterone. In the short time-course, CaMKII phosphorylation increased at 5 min and peaked at 15 min after testosterone stimulation (**Figure 1A**), whereas in the longer time-course, CaMKII phosphorylation peaked at 3 h, to a level lower than that at 15 min, and then returned to its basal level within 6–9 h (**Figure 1B**).

Because the maximal effect of testosterone on CaMKII phosphorylation was reached at 15 min after stimulation, we assessed the CaMKII phosphorylation at 15 min in response to stimulation of cardiac myocytes with 1, 50, 100, 500, or 1000 nM testosterone: CaMKII phosphorylation was increased at 50 nM and was maximally elevated at 100 nM, with higher concentrations of testosterone being relatively less effective in increasing CaMKII phosphorylation (**Figure 1C**). To test whether this increase in phosphorylation was associated with enhanced CaMKII activity, we measured the levels of Thr17 phosphorylation in PLN, a downstream target for activated CaMKII (Mattiazzi et al., 2005). Testosterone (100 nM) increased PLN phosphorylation and the level peaked at 30 min (**Figure 1D**).

### Testosterone Increases MEF2 Activity in Cardiac Myocytes

**FIGURE 2:**
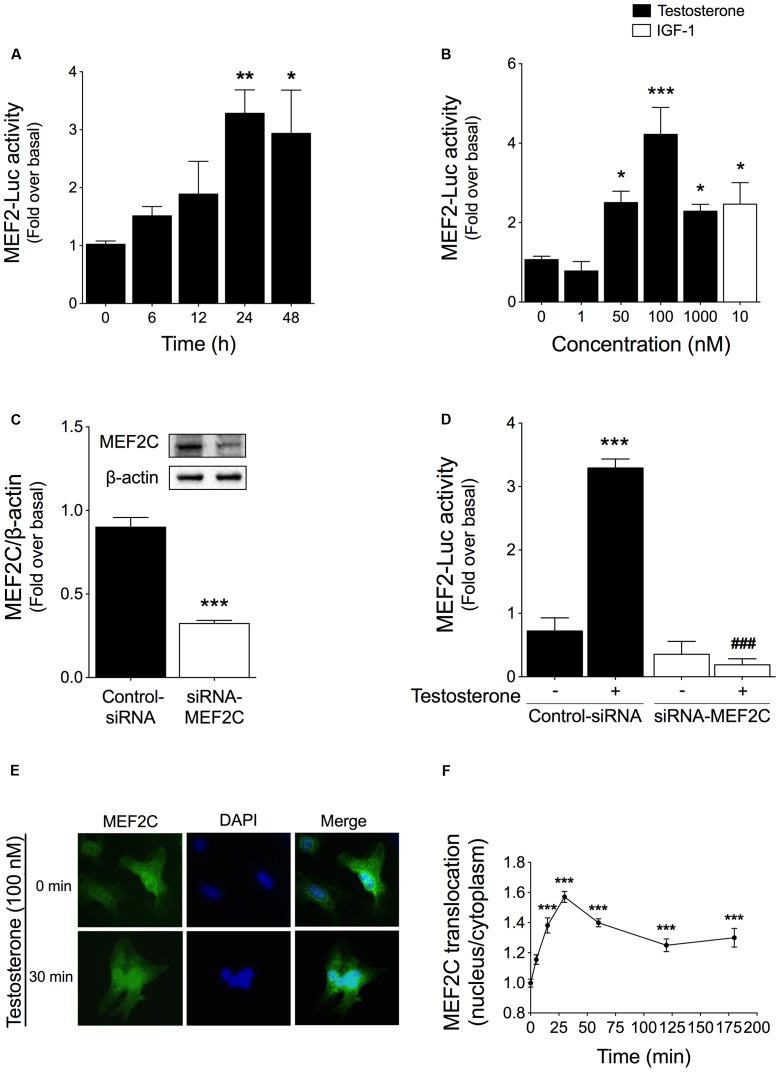
Testosterone-induced MEF2 activity in cardiac myocytes. Cells were transfected with MEF2 luciferase-reporter (MEF2-Luc) and *Renilla* luciferase plasmids. MEF2 activity is expressed as MEF2-Luc to *Renilla* luciferase ratio. **(A)** Cardiac myocytes were stimulated with 100 nM testosterone for 6–48 h (*n* = 6). **(B)** Cells were treated with testosterone at the indicated concentrations for 24 h (*n* = 6). IGF-1 treatment (10 nM, 24 h) was used as the positive control for MEF2 activity (*n* = 6). **(C)** Cardiac myocytes were transfected with either siRNA-MEF2C or non-targeting siRNA. **(D)** Cardiac myocytes expressing MEF2-Luc were transfected with siRNA-MEF2C and stimulated with testosterone (100 nM) for 24 h (*n* = 5). Cells transfected with the non-targeting siRNA served as the control. **(E)** Cells were stimulated with testosterone (100 nM) for 5–180 min and then subjected to immunofluorescent staining with an anti-MEF2C antibody; nuclei were stained with DAPI (blue). The figure shows representative images for control and stimulated conditions (30 min). **(F)** Quantification of MEF2C staining, shown as the nuclear-to-cytoplasmic fluorescence ratio. Data are presented as means ± SEM or as representative images. *P*-values were determined using *t*-test or ANOVA followed by Bonferroni *post hoc* test. ^∗^*P* < 0.05, ^∗∗^*P* < 0.01, ^∗∗∗^*P* < 0.001 vs. control; ^###^*P* < 0.001 vs. testosterone.

The transcription factor MEF2 is a crucial downstream target that integrates CaMKII signaling pathways (Zhang et al., 2007). To investigate the ability of testosterone to stimulate MEF2 activity, cardiac myocytes were transfected with a MEF2 luciferase reporter plasmid (MEF2-Luc) and then stimulated with 100 nM testosterone for 6, 12, 24, or 48 h. Testosterone-stimulated MEF2 activity peaked at 24 h and was sustained for at least 48 h (**Figure 2A**). Next, we evaluated MEF2 activity at 24 h after stimulation with different concentrations of testosterone (1, 50, 100, or 1000 nM), which revealed that maximal MEF2 activity was stimulated by 50–100 nM testosterone (**Figure 2B**); here, treatment with 10 nM IGF-1 for 24 h served as the positive control for MEF2 activation in cardiac myocytes. Moreover, specific activation of MEF2C by testosterone was confirmed knocking down MEF2C protein using RNA interference. Transfection of siRNA-MEF2C reduced MEF2C protein expression by 63% as compared to transfection with a negative-control siRNA (**Figure 2C**). Downregulation of MEF2C protein abolished the increase in MEF2 activity measured in response to testosterone treatment of cardiac myocytes (**Figure 2D**).

MEF2 activity and MEF2C nuclear localization are increased by IGF-I, which is a pro-hypertrophic hormone (Munoz et al., 2009). To determine whether testosterone induces nuclear translocation of MEF2C, we evaluated the subcellular distribution of MEF2C protein in cardiac myocytes. After testosterone stimulation, MEF2C immunofluorescence was localized mainly in the nucleus, as evidenced by the overlap with DAPI counterstaining (**Figure 2E**). Furthermore, quantification of the fluorescence intensity of MEF2C staining in the nucleus and the cytoplasm (**Figure 2F**) revealed that the nuclear-to-cytoplasmic fluorescence ratio increased and peaked at 30 min after testosterone stimulation, which indicated nuclear translocation of MEF2C protein.

### Testosterone Activation of MEF2 Is Mediated Through CaMKII

**FIGURE 3:**
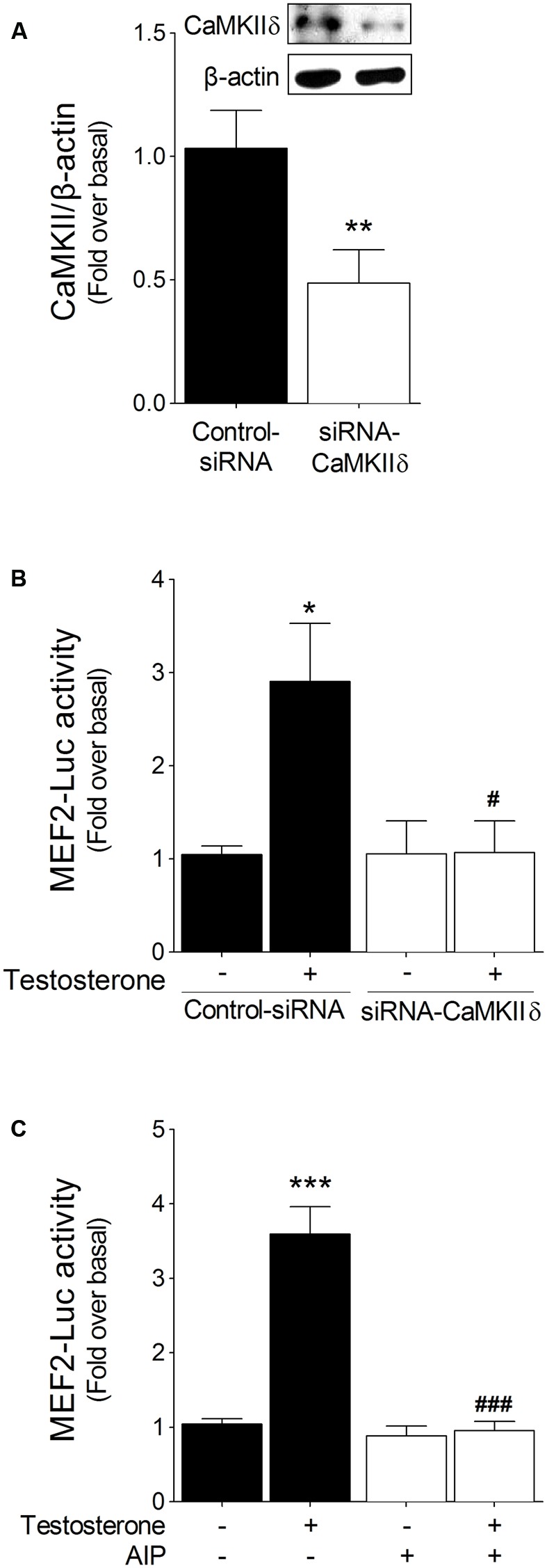
MEF2 activation by testosterone is mediated through CaMKII. **(A)** Cardiac myocytes were transfected with siRNA-CaMKIIδ (20 nM) or a non-targeting siRNA as a control and checked for CaMKII expression. **(B)** Cells transfected with the siRNAs and MEF2-Luc were stimulated with testosterone (100 nM) for 24 h, after which MEF2 activity was measured (*n* = 5). **(C)** Cells transfected with MEF2-Luc were incubated with autocamtide-2-related inhibitory peptide (AIP, 1 μM) for 30 min and then stimulated with testosterone (100 nM) for 24 h (*n* = 6). Data are presented as means ± SEM. *P*-values were determined using *t*-test or ANOVA followed by Bonferroni *post hoc* test; ^∗∗^*P* < 0.01, ^∗∗∗^*P* < 0.001 vs. control; ^#^*P* < 0.05, ^###^*P* < 0.001 vs. testosterone.

To study whether MEF2 activation by testosterone is mediated through CaMKII, we either transfected cardiac myocytes with siRNA-CaMKIIδ, the principal CaMKII isoform expressed in heart (Gray and Heller Brown, 2014), or pretreated the cells with 1 μM AIP, a cell-permeable peptide inhibitor of CaMKII. Transfection with siRNA-CaMKIIδ resulted in a 52% reduction in CaMKIIδ protein content in cardiac myocytes (**Figure 3A**), and, notably, CaMKIIδ downregulation abolished the activation of MEF2 induced by testosterone (**Figure 3B**). Similarly, CaMKII inhibition by AIP blocked the testosterone-induced increase in MEF2 activity (**Figure 3C**). These results suggest that CaMKII functions upstream of MEF2 in testosterone-triggered MEF2 activation in cardiac myocytes.

### AR and CaMKII Are Involved in Testosterone-Induced MEF2 Activation

**FIGURE 4:**
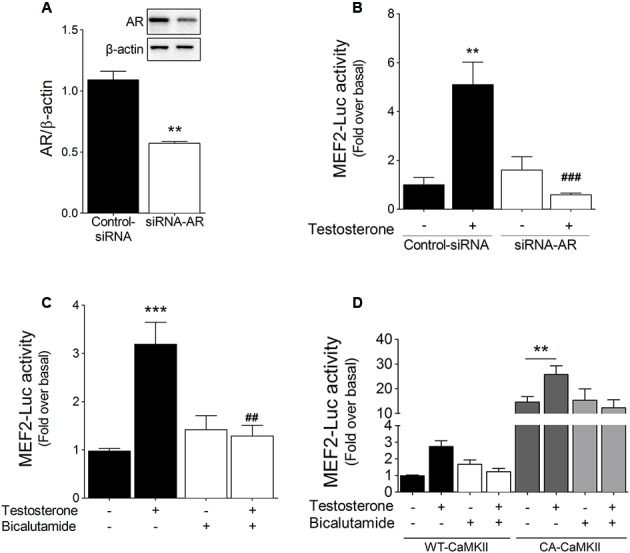
AR and CaMKII modulate testosterone-induced MEF2 activation. **(A)** Cardiac myocytes were transfected with siRNA-AR (20 nM) or control non-targeting siRNA. **(B)** Cardiac myocytes expressing MEF2-Luc reporter were transfected with siRNA-AR and stimulated with 100 nM testosterone for 24 h (*n* = 5). **(C)** Cardiac myocytes expressing MEF2-Luc were treated with bicalutamide (1 μM, 30 min) and then stimulated with testosterone, after which MEF2-Luc activity was measured. **(D)** Cardiac myocytes were transfected with either wild-type (WT) or constitutively active (CA) CaMKII and then stimulated with testosterone for 24 h in the presence or absence of bicalutamide (1 μM). Data are presented as means ± SEM. *P*-values were determined using *t*-test or ANOVA followed by Bonferroni *post hoc* test; ^∗∗^*P* < 0.01, ^∗∗∗^*P* < 0.001 vs. control; ^##^*P* < 0.01, ^###^*P* < 0.001 vs. testosterone.

To assess the role of AR in testosterone-induced MEF2 activity, cardiac myocytes were transfected with siRNA-AR. In these transfected cells, AR protein expression was decreased by ∼49% (**Figure 4A**), and, furthermore, testosterone-stimulated increase in MEF2 activity was abolished (**Figure 4B**). Similar results were obtained in cells pretreated with the AR inhibitor bicalutamide (1 μM) (**Figure 4C**).

Next, we investigated whether MEF2 activation by testosterone links the CaMKII and AR signaling pathways. To evaluate the roles of CaMKII and AR in the activation of MEF2, cardiac myocytes were transfected with either constitutively active CaMKII (CA-CaMKII) or wild-type CaMKII (WT-CaMKII). As compared to WT-CaMKII overexpression, CA-CaMKII overexpression increased the basal activity of MEF2, and subsequent stimulation with 100 nM testosterone resulted in an additional increase in MEF2 activity (**Figure 4D**). Moreover, we found that bicalutamide pretreatment of CA-CaMKII-expressing cardiac myocytes did not affect the elevation of MEF2 basal activity, but it prevented the further activity increase induced by testosterone (**Figure 4D**). Collectively, these results suggest that CaMKII and AR signaling pathways regulate MEF2 activation in response to testosterone in cardiac myocytes.

### Testosterone-Induced Cardiac Myocyte Hypertrophy Involves CaMKII/MEF2 and AR Signaling Pathways

**FIGURE 5:**
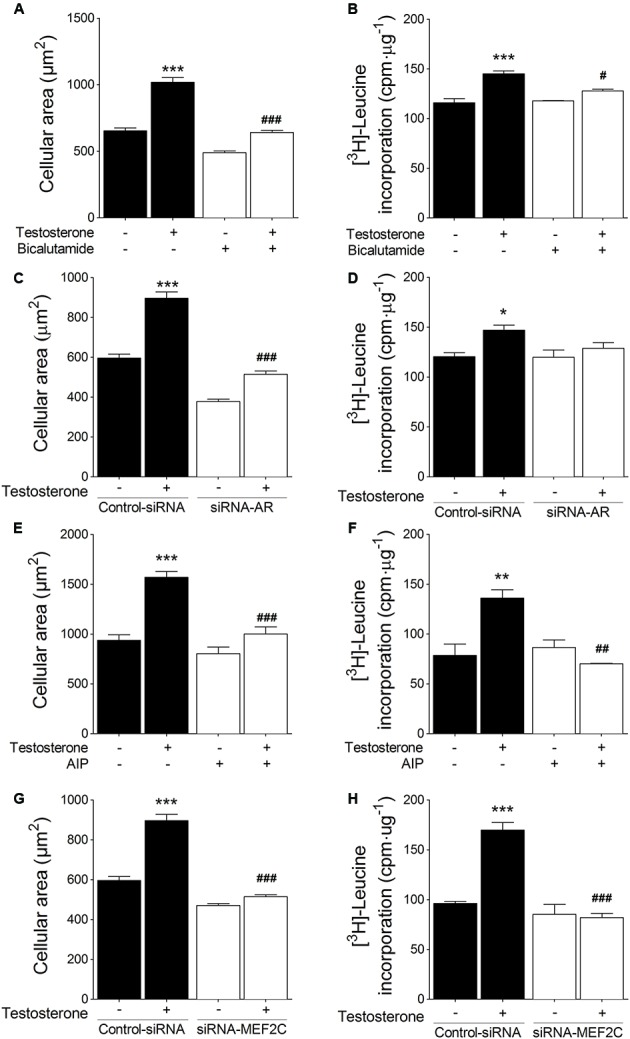
Effect of inhibition of CaMKII, MEF2C, and AR on testosterone-induced cardiac myocyte hypertrophy. Cell area and [^3^H]-leucine incorporation were evaluated as hypertrophy parameters. Cells were treated with 100 nM testosterone for 48 h after **(A,B)** pretreatment with 1 μM bicalutamide, **(C,D)** transfection with siRNA-AR, **(E,F)** pretreatment with 1 μM AIP, or **(G,H)** transfection with siRNA-MEF2C. Cellular area was assessed using the vital fluorescent dye CellTracker Green (*n* = 100 cells per condition from 5 independent cultures). Incorporation of [^3^H]-leucine was quantified using a liquid scintillation counter, and values are expressed as counts⋅min^-1^⋅(μg of protein)^-1^ with respect to control non-stimulated conditions (*n* = 5). Data are presented as means ± SEM. *P*-values were determined using ANOVA followed by Bonferroni *post hoc* test; ^∗^*P* < 0.05, ^∗∗^*P* < 0.01, ^∗∗∗^*P* < 0.001 *vs*. control; ^#^*P* < 0.05, ^##^*P* < 0.01, ^###^*P* < 0.001 vs. testosterone.

To examine the involvement of CaMKII/MEF2 and AR signaling pathways in testosterone-induced cardiac hypertrophy, we measured cellular size and [^3^H]-leucine incorporation, which are both accepted indicators of cardiac myocyte hypertrophy. First, we determined whether hypertrophy depends on AR activation: we either preincubated cardiac myocytes with 1 μM bicalutamide or transfected the cells with siRNA-AR. Stimulation with testosterone (100 nM) for 48 h increased cardiac myocyte size and [^3^H]-leucine incorporation, and both of these effects were blocked by bicalutamide (**Figures 5A,B**) and siRNA-AR (**Figures 5C,D**). Second, when cardiac myocytes were treated with 1 μM AIP, the CaMKII inhibitor, the testosterone-induced increase in both cardiac myocyte size and [^3^H]-leucine incorporation was blocked (**Figures 5E,F**). Furthermore, MEF2C downregulation mediated by siRNA-MEF2C abolished cardiac myocyte hypertrophy stimulated by testosterone (**Figures 5G,H**). These results together suggest that testosterone links the CaMKII signaling pathway to MEF2C- and AR-regulated transcription in cardiac myocyte hypertrophy.

### CaMKII, MEF2C, and AR Pathways Function in Testosterone-Induced Cardiac Hypertrophy *In Vivo*

**FIGURE 6:**
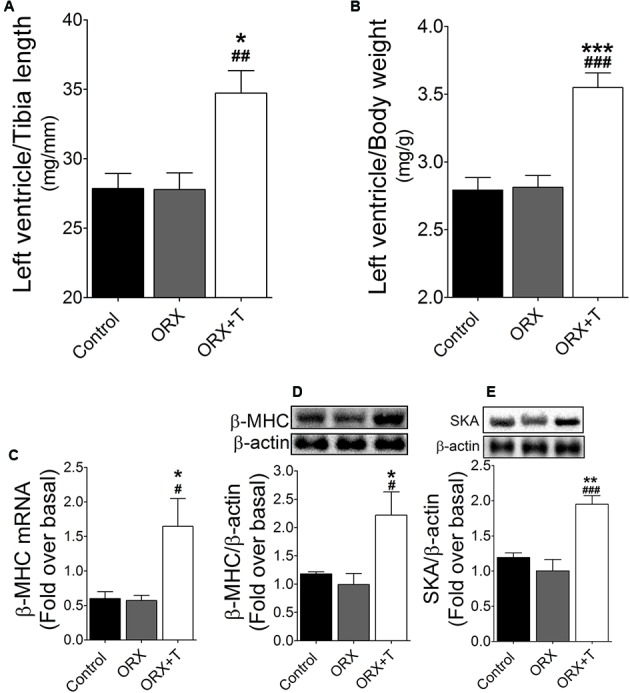
Cardiac hypertrophy induced by testosterone *in vivo*. ORX rats were treated with testosterone (ORX + T) or vehicle (ORX). Normal rats were used as control group. In these animals, the following ratios were determined: **(A)** Left-ventricle weight/body weight; **(B)** Left-ventricle weight/tibia length. **(C)** β-MHC mRNA levels were assessed using qPCR (*n* = 6 rats per experimental condition). The mRNA levels of these hypertrophy marker genes were normalized relative to GAPDH mRNA expression, and the values shown here correspond to target-gene/GAPDH mRNA ratios. **(D)** β-MHC and **(E)** SKA protein levels were determined by western blot. The results of densitometric analyses are shown as ratios of β-MHC or SKA protein levels normalized to β-actin as protein loading control. Data are presented as means ± SEM; *P*-values were determined using ANOVA followed by Bonferroni *post hoc* test; ^∗^*P* < 0.05, ^∗∗^*P* < 0.01, ^∗∗∗^*P* < 0.001 vs. control; ^#^*P* < 0.05, ^##^*P* < 0.01, ^###^*P* < 0.001 vs. ORX group.

Lastly, we conducted *in vivo* studies to investigate the physiological relevance of the findings obtained using cultured cardiac myocytes: we examined the function of the CaMKII/MEF2 and AR signaling pathways in control and ORX rats that were or were not administered testosterone. In the ORX rats treated with testosterone, the following ratios were increased: left-ventricle weight/body weight (**Figure 6A**) and left-ventricle weight/tibia length (**Figure 6B**). Plasma testosterone concentrations were significantly higher in the testosterone-treated rats than in untreated rats, and, furthermore, demonstrating that testosterone produced its expected effects in this *in vivo* hypertrophy model, the weights of seminal vesicles and prostates (which are sensitive indicators of testosterone actions) were markedly higher in the testosterone-administered ORX rats than in ORX or control rats (**Table 1**). Moreover, testosterone supplementation upregulated β-MHC mRNA expression (**Figure 6C**) as well as β-MHC and SKA protein levels compared with ORX or control rats (**Figures 6D,E**).

**Table 1:** Animal characteristics and hormone concentrations.

	Control	ORX	ORX + T
	
*n*	6	6	6
Testosterone concentration (ng⋅ml^-1^)	1.87 ± 0.3	0.14 ± 0.01^∗^	44.01 ± 5.5^∗∗^ ###
Body weight (g)	448.3 ± 7.4	499.3 ± 12.1^∗^	440.2 ± 14.7 ##
Seminal vesicle weight (g)	1.44 ± 0.05	0.16 ± 0.01^∗∗∗^	1.58 ± 0.12 ###
Prostate weight (g)	1.22 ± 0.09	0.56 ± 0.07^∗∗∗^	2.77 ± 0.14^∗∗^ ###

*ORX, orchiectomized rats; T, testosterone. Values are means ± SEM; P-values were determined using ANOVA followed by Bonferroni post hoc test; ^∗^P < 0.05, ^∗∗^P < 0.01, ^∗∗∗^P < 0.001 vs. control; #P < 0.05, ##P < 0.01, ###P < 0.001 vs. ORX group*.

**FIGURE 7:**
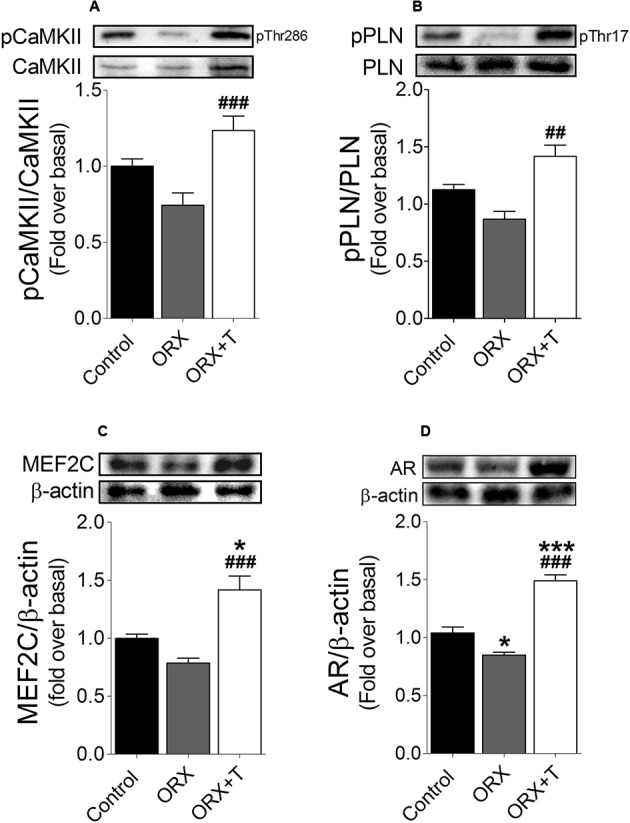
Testosterone increases CaMKII activity and MEF2C and AR protein expression in cardiac hypertrophy *in vivo.* Extracts of homogenized left-ventricle tissue from the different rat groups were subjected to western blot to measure **(A)** CaMKII phosphorylation at Thr286 and total CaMKII protein levels, and **(B)** PLN Thr17 phosphorylation and total protein levels (*n* = 6); in these two panels, the values shown are phosphorylated-to-total protein ratios. **(C)** MEF2C and **(D)** AR protein levels were determined through western blotting; β-actin was used as the loading control. Data are presented as means ± SEM; *P*-values were determined using ANOVA followed by Bonferroni *post hoc* test; ^∗^*P* < 0.05, ^∗∗^*P* < 0.01, ^∗∗∗^*P* < 0.001 vs. control; ^#^*P* < 0.05, ^##^*P* < 0.01, ^###^*P* < 0.001 vs. ORX group.

Additionally, we determined the phosphorylation levels of CaMKII (Thr286) and PLN (Thr17) by immunoblotting extracts of left-ventricle tissue from testosterone-administered ORX and control rats. Testosterone increased the phosphorylation of both CaMKII (**Figure 7A**) and PLN (**Figure 7B**). Moreover, in the testosterone-treated ORX rats, MEF2C and AR protein levels were higher than untreated ORX or control rats (**Figures 7C,D**).

## Discussion

This study provided new insights into the mechanisms of testosterone action in cardiac myocytes, revealing the involvement of distinct—but interlinked—signal transduction and transcription pathways: Our findings presented here support a functional link between CaMKII/MEF2 signaling and AR-mediated transcription in cardiac myocyte hypertrophy induced by testosterone.

Currently, cell-specific testosterone effects are considered to involve the activation of both AR-dependent and -independent signal transduction pathways (Bolton et al., 2007; Heemers and Tindall, 2007; Kadi, 2008; Altamirano et al., 2009; Kloner et al., 2016). Previously, we showed that testosterone induced an increase in CaMKII phosphorylation in <30 min in cardiac myocytes (Wilson et al., 2013). Here, analysis of CaMKII phosphorylation kinetics over short and long-time courses in testosterone-stimulated cardiac myocytes revealed that CaMKII phosphorylation peaked mainly at 15 min and then to a comparatively lower level at 3 h post-stimulation. Testosterone also increased PLN phosphorylation at Thr17, which supports the conclusion that CaMKII was fully activated (Mattiazzi et al., 2005). These results agree with those reported previously in cardiac tissue (Witayavanitkul et al., 2013; Vutthasathien and Wattanapermpool, 2015). CaMKII activity is regulated through Ca^2+^ signals, and testosterone induces intracellular Ca^2+^ increase in cardiomyocytes. In a previous work, we showed that testosterone increase intracellular Ca^2+^ levels in a concentration-dependent manner peaking at 100 nM and higher concentration did not alter these Ca^2+^ signals (Vicencio et al., 2006). Several reports demonstrated that different pattern of Ca^2+^ signals modulate CaMKII. In the present study, we cannot rule out that high levels of testosterone induce other intracellular pathways which might regulate either positive- or negatively the activity of CaMKII in cardiac myocytes.

Testosterone treatment induced MEF2 activity and the nuclear translocation of MEF2C, and the downregulation of MEF2C eliminated this MEF2 activity, which indicated specific activation of the MEF2C isoform. The *in vivo* importance of the MEF2 pathway in the development of cardiac hypertrophy has been examined in hearts from rats subjected to pressure overload (Molkentin and Markham, 1993) and in transgenic mice overexpressing CaMKIIδ, MEF2 activity was increased (Zhang et al., 2007). Another key finding of our study was that the increase in MEF2 activity induced by testosterone was mediated by CaMKII in cultured cardiac myocytes. Downregulation of CaMKIIδ protein expression or treatment of cells with a cell-permeable peptide inhibitor of CaMKII abolished testosterone-induced MEF2 activation, which indicates that CaMKII is an upstream regulator of MEF2 activity. CaMKII regulates MEF2 activity by phosphorylation and nuclear residence via HDAC4/5 (Backs et al., 2008). Several reports indicate that dynamic CaMKII-dependent regulation can induce longer-term effects on its downstream target in cardiac cells (Wu et al., 2006). Furthermore, overexpression of CA-CaMKII (CaMKIIT286D) increased basal MEF2 activity in cardiac myocytes, which agrees with previous results obtained *in vivo* (Zhang et al., 2007). In cardiac myocytes expressing CaMKIIT286D, testosterone stimulation produced an additional increase in MEF2 activity, but this increase was eliminated following treatment with bicalutamide, which suggests that an AR-dependent mechanism participates in the activation. Moreover, the actions of MEF2 through CaMKII signaling may involve additional downstream transcriptional pathways to control testosterone-induced cardiac hypertrophy.

To investigate functional relevance of the findings that we obtained using cultured cardiac myocytes, we examined the aforementioned signaling pathways in an *in vivo* model of testosterone-induced cardiac hypertrophy. Our results showed that in the hypertrophied hearts obtained from testosterone-supplemented ORX rats, CaMKII phosphorylation at Thr286 and PLN phosphorylation at Thr17 were increased as compared with the levels in vehicle-treated ORX or control rats. These results agree with the findings of Witayavanitkul et al. (2013) who reported that PLN phosphorylation at Thr17 was decreased in castrated rats but was restored following testosterone administration, which suggested that testosterone levels regulate CaMKII and PLN phosphorylation *in vivo*. Furthermore, we found that in left-ventricle tissue extracts from testosterone-supplemented ORX rats, MEF2C and AR protein levels were increased relative to untreated ORX or control animals. A recent report suggests that administration of testosterone 20 mg⋅kg-1 by 4 weeks induce physiological cardiac hypertrophy, but prolonged treatment by 12 weeks become to be detrimental and cause pathological cardiac hypertrophy (Pirompol et al., 2016). Moreover, another study suggests that high concentration of testosterone (125 mg⋅kg-1 for 4 weeks) increases hypertrophy markers, but did not change infarction size and mortality after coronary artery ligation and hemodynamic parameters(Nahrendorf et al., 2003). Collectively, these data suggest that testosterone integrates multiple signaling pathways *in vivo*.

AR-mediated transcription of target genes is regulated in a ligand-inducible manner (Bolton et al., 2007; Claessens et al., 2008). Several transcription factors interact with and influence the transcriptional activity of androgens, including GATA, STAT5, NF1, and SP1 (Heemers and Tindall, 2007). The coregulators that influence AR transcriptional activity either interact at ARE binding sites or assemble functional protein-protein complexes in the promoter regions of the target genes (McEwan, 2004; Bolton et al., 2007; Heemers and Tindall, 2007). In skeletal muscle, testosterone upregulates the expression of genes involved in sarcolemma integrity, contraction, metabolism, and cell growth (Kadi, 2008; Wyce et al., 2010). Moreover, the induction of cardiac hypertrophy is regulated predominantly at the transcriptional level (Akazawa and Komuro, 2003). Recent evidence suggested that testosterone links MEF2C to AR-regulated transcription (Bagchi et al., 2011), and, notably, AR-binding regions were reported to be enriched in MEF2-binding sequences, and MEF2C-dependent genes were also identified as targets for AR signaling, which suggests a functional interaction of these two transcription factors in skeletal muscle (Wyce et al., 2010). Furthermore, testosterone was shown to stimulate cardiac myocyte differentiation from mouse embryonic stem cells and P19 embryonic carcinoma cells through AR-regulated transcription involving ARE regions of MEF2C-regulated genes, which led to an increase in histone acetylation in the DNA (Di-Luoffo et al., 2015), and the MADS domains of MEF2 were reported to potentially generate regulatory protein–protein interactions for MEF2C and AR (Black and Olson, 1998). Thus, the differential expression of testosterone-regulated genes entails the integration of transcriptional modulators (Verrijdt et al., 2003; McEwan, 2004; Heemers and Tindall, 2007). Accordingly, NFAT-controlled gene transcription was found to involve additional transcription factors, including MEF2 (Cortes et al., 2012). Recently, we determined that testosterone stimulates cardiac myocyte hypertrophy through NFAT activation and GSK-3β inhibition, which supports a cooperative mechanism involving cytoplasmic and nuclear signaling (Duran et al., 2016).

It has been described that pro-hypertrophic agents induce nuclear translocation and activation of GRK, which modulates MEF2 in cardiac cells (Martini et al., 2008; Gold et al., 2013). It has been reported that aldosterone-induced cardiac hypertrophy involves MEF2 activation through GRK5, one of the main isoforms of GRK found in heart. The mechanism depends of GRK5 nuclear translocation, binding to calmodulin and the phosphorylation of MEF2-repressive protein HDAC5, together with non-canonical actions involving the mineralocorticoid receptor and AT1R (Cannavo et al., 2016). Aldosterone-induced fibrosis and apoptosis in a GRK5-dependent mechanism, suggesting an involvement of steroids in cardiac hypertrophy and apoptosis (Cannavo et al., 2016). However, there are few studies reporting hypertrophic and apoptotic effects of androgens. Whereas an article reports that elevated concentrations of androgens induce apoptosis in the H2C9 cardiomyocyte cell line (do Nascimento et al., 2015), or prevent apoptosis induced by cardiotoxic agents in an AR dependent mechanism (Ikeda et al., 2010), another study indicates that cardiac apoptosis in castrated animals was reduced by testosterone administration (Kang et al., 2012). Moreover, Ikeda et al. (2012) reported that doxorubicin treatment in Androgen Receptor KO mice, which exhibit impaired production of testicular androgens, show higher levels of apoptosis and oxidative stress markers than control mice (Ikeda et al., 2012). These results suggest that androgen signaling may be cardioprotective and anti-apoptotic in response to cardiotoxic agents.

Androgens modulate the metabolism, differentiation, and growth of cardiac myocytes, but the underlying mechanisms and the functional interactions in testosterone signaling in cardiac myocytes remain poorly defined. To further extend testosterone-replacement therapies effectively, comprehensive approaches must be used to elucidate the mechanisms that operate during the shift of cardiac myocytes from a normal to a hypertrophied state. The cell signaling network described in this study suggests that testosterone-induced cardiac hypertrophy integrates cytoplasmic and nuclear signaling pathways.

## Author Contributions

ME and JD conceived and designed the studies and wrote the first draft of the manuscript. JD, DL, MP, SR, MT, GB, SL, and ME performed the experiments, biochemical studies, and analyzed the data. GB, CI, and SL contributed to the study and experimental design and reviewed the manuscript. JD and ME wrote the paper. All authors approved the final version for submission.

## Conflict of Interest Statement

The authors declare that the research was conducted in the absence of any commercial or financial relationships that could be construed as a potential conflict of interest.

## Abbreviations

AIP: autocamtide-2-related inhibitory peptide
ARE: Androgen response element
β-MHC: β-myosin heavy chain
CaMKII: Ca^2+^/calmodulin-dependent protein kinase II
GRK: G protein-coupled receptor kinase
MADS: MCM-1, Agamous, Deficiens, Serum response factor
MEF2: myocyte-enhancer factor 2
ORX: orchiectomized
PLN: Phospholamban
SKA: α-skeletal actin
